# Human Cytomegalovirus-Encoded miR-US25-1 Aggravates the Oxidised Low Density Lipoprotein-Induced Apoptosis of Endothelial Cells

**DOI:** 10.1155/2014/531979

**Published:** 2014-05-08

**Authors:** Jianmin Fan, Wen Zhang, Qiming Liu

**Affiliations:** ^1^The First Affiliated Hospital of Hunan University of Traditional Chinese Medicine, Changsha 410007, China; ^2^Cardiovascular Medicine Department, The Second Xiangya Hospital of Central South University, Changsha 410011, China

## Abstract

Human cytomegalovirus (HCMV) infection is linked to the development and severity of the cardiovascular disease atherosclerosis; however, there is little known about the promotion of atherosclerosis. miR-US25-1 is one of HCMV-encoded miRNAs and targets cellular genes that are essential for virus growth to control the life cycle of the virus and host cells. The prominent regulation on cell cycle genes of the miR-US25-1 attracts us to explore its role in the atherosclerosis promotion. It was indicated that miR-US25-1 level was upregulated in subjects or in endothelial cells with HCMV infection; and the miR-US25-1 downregulated the expression of BRCC 3 by targeting the 5′ UTR of BRCC 3. And a miR-US25-1 mimics transfection could reduce the EAhy926 cell viability but did not induce apoptosis in EAhy926 cells. And what is more, miR-US25-1 mimicis transfection deteriorated the ox-LDL-induced apoptosis and aggravated the upregulation of apoptosis-associated molecules by oxidised low density lipoprotein (ox-LDL) in EAhy926 cells. And we have also confirmed the deregulation of BRCC 3 expression by miR-US25-1 by targeting the 5′ UTR of it. Given the vital role of BRCC 3 in DNA damage repairing, we speculated that the targeting inhibition of BRCC 3 by miR-US25-1 may contribute to the aggravation of ox-LDL-promoted apoptosis of endothelial EAhy926 cells.

## 1. Introduction

It is well known that the oxidized low density lipoprotein (ox-LDL) plays a key role in the development of atherosclerosis [1]. And multiple types of cells, such as endothelial cells, macrophages, and smooth muscle cells, are involved in the ox-LDL-promoted atherosclerosis [2]. Ox-LDL is considered to induce apoptosis, monocyte adhesion, and reactive oxygen species generation [3–5] via upregulating [4] and binding to the lectin-like endothelial ox-LDL receptor (LOX-1) [4, 6] on the vascular endothelial cells. And various molecules play roles in the ox-LDL-induced apoptotic cascade, such as caspases [6], AIF [7], VPO1 [8], PKC, PTK, bcl-2, and Fas [9]. However, other studies show converse results. Prior exposure to ox-LDL limits apoptosis in subsequent generations of endothelial cells by altering promoter methylation [10].

The sustained high level of ox-LDL will finally lead to atherosclerosis. And what is more, there is a key role in the atherosclerosis acceleration by infection and inflammation [11–14]. The inflammation in vascular system is caused by vessel wall injury and endothelial cell (EC) dysfunction [15, 16] and is triggered by infectious agents such as human cytomegalovirus (HCMV) [17, 18]. Then, the following monocyte activation and cytokine and chemokine overproduction promote and accelerate the atherosclerotic plaque formation, endothelial and smooth muscle cell proliferation, atherosclerotic plaque rupture, and thrombus formation [19–23]. HCMV infection is linked to the development and severity of the cardiovascular disease atherosclerosis [24]. HCMV has clearly been shown to be associated with an enhanced rate of restenosis and vasculopathy [25, 26]. Additionally, serological studies indicate a link between HCMV and atherosclerosis [27, 28]. Most knowledge about the molecular and cellular bases for the pathogenic effects of HCMV is based on its influence on the pattern of host cell gene expression [17, 29]. Different molecules have been identified to be mediating the HCMV-induced changes of the cellular response including cytokines [30] and growth factors [31]. Up to now, it is not clear whether structural or no structural molecules expressed by HCMV are directly involved in the promotion of atherosclerosis.

MicroRNAs (miRNAs) are endogenous, noncoding RNA molecules of 18–22 nt that can bind the 3′-untranslated region of target messenger RNA (mRNA) and regulate gene expression in a broad array of cell processes in mammals [32–35]. And the regulation of miRNAs in the cardiovascular system has also been well confirmed [36–38]. Herpesviruses belong to a large family of enveloped, double-stranded DNA viruses that are able to maintain a persistent or latent infection during the lifetime of the virus in its host. Belonging to one of the three groups of herpesvirus, HCMV has been shown to encode miRNAs, indicating that HCMV has utilized the RNA interference machinery throughout their evolution [39]. HCMV miRNAs are spread throughout the viral genome and have been demonstrated to be expressed during acute lytic infection [40–43]. miR-US25-1 is one of HCMV-encoded miRNAs and targets cellular genes that are essential for virus growth to control the life cycle of the virus [44]. More recently, it is shown that the viral miR-US25-1 downregulates multiple cell cycle genes through mRNA 5′ UTRs [45]. The prominent regulation of cell cycle genes of the miR-US25-1 attracts us to explore its role in the atherosclerosis promotion.

The present study revealed that human cytomegalovirus-encoded miR-US25-1 aggravates the ox-LDL-induced apoptosis of endothelial cells via targeting and downregulating BRCC 3.

## 2. Results

### 2.1. Upregulation of miR-US25-1 Level in Subjects or in Endothelial Cells with HCMV Infection

**Figure 1:**
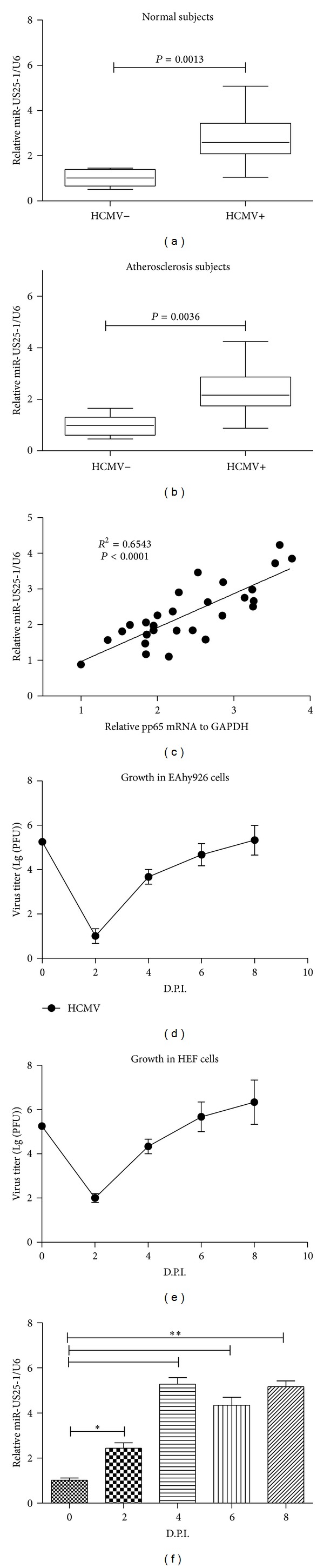
HCMV infection promoted miR-US25-1 level* in vivo* or* in vitro.* (a) The expression of miR-US25-1 was examined in blood samples of normal subjects with (*n* = 28) or without (*n* = 10) HCMV positive. (b) The expression of miR-US25-1 was examined in blood samples of atherosclerosis subjects with (*n* = 32) or without (*n* = 12) HCMV positive. (c) Correlation of miR-US25-1 expression with plasma pp65 mRNA level. (d) and (e) Growth curve of HCMV in EAhy926 or HEF cells, MOI = 0.1. (f) HCMV infection upregulates miR-US25-1 level in EAhy926 cells. All experiments for (d)–(f) were performed in triplicate independently. Statistical significance was showed as *P < 0.05 and **P < 0.01.

miR-US25-1 has been well confirmed to be encoded by HCMV to control the life cycle of the virus [44]; we detected the miR-US25-1 level (U6, the most highly conserved small nuclear RNA across species [46], as internal control) in whole blood samples of normal subjects with or without HCMV positive. It was indicated that there was a significant upregulation of the miR-US25-1 in whole blood of HCMV associated subjects (2.72 ± 1.13 versus 1.00 ± 0.35 in HCMV-negative subjects, *P* = 0.0013) (Figure 1(a)). A significant upregulation of blood miR-US25-1 level was also confirmed in the atherosclerosis subjects with HCMV positive. As show in Figure 1(b), the average value of miR-US25-1 level in HCMV-infected atherosclerosis subjects was 2.31 ± 0.71, significantly higher than 1.00 ± 0.42 in the HCMV-negative atherosclerosis subjects (*P* = 0.0036). To further confirm the association of miR-US25-1 level with the active HCMV infection, the correlation between miR-US25-1 expression and the mRNA level of pp65, one of HCMV tegument proteins in the atherosclerosis patients, was explored.  Figure 1(c) indicated a significant correlation of miR-US25-1 level with the pp65 mRNA level (*R* ^2^ = 0.6543, *P* < 0.0001).

To evaluate the miR-US25-1 expression in endothelial cells after HCMV infection, we determined the growth curve of the virus in EAhy926 cells with a control of virus growth curve in HEF cells. It was shown that HCMV replicated well in EAhy926 cells, the virus titer elevated to 5.33 (Lg (PFU/mL)) at eight days after inoculation, with a similar growth curve in HEF cells, though significantly less than the titer in HEF cells (Figures 1(d) and 1(e)). And then the miR-US25-1 expression in EAhy926 cells was also determined; Figure 1(f) demonstrated a significant high miR-US25-1 level in EAhy926 cells from 2 to 8 D.P.I. compared with that in 0 D.P.I. (*P* < 0.05 in 2 D.P.I. and *P* < 0.01 in 4, 6, or 8 D.P.I., resp.). Taken together, the* in vivo* and* in vitro* results confirmed the upregulation by HCMV infection.

### 2.2. miR-US25-1 Reduces Cell Viability but Does Not Induce Apoptosis in EAhy926 Cells

**Figure 2:**
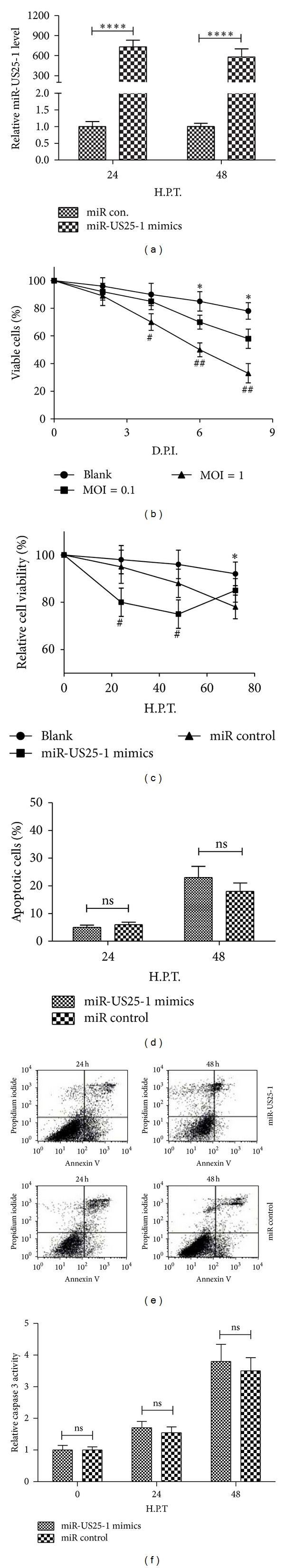
miR-US25-1 mimics reduced EAhy926 cell viability but did not induce apoptosis in EAhy926 cells. (a) The miR-US25-1 level was promoted by miR-US25-1 mimics, rather than miR control, both of which were transfected in EAhy926 cells with 50 nM. (b) Viability of EAhy926 cells decreased after HCMV infection with 0.1 or 1 MOI (compared to blank), revealed by MTT assay. (c) miR-US25-1 mimics, rather than miRNA control, reduced EAhy926 cell viability, revealed by MTT assay. (d) No significant difference in regulation of apoptosis of EAhy926 cells by the miR-US25-1 mimics and the miRNA control. The results are expressed as percentages of positive mean values ± S.E. for three independent experiments. (e) Apoptosis of EAhy926 cells with miR-US25-1 mimics or miRNA control transfection of 50 nM. (f) No significant difference in caspase 3 activity in EAhy926 cells after miR-US25-1 mimics or miRNA control transfection. ns: no significance, ^#^ or *P < 0.05, ^##^ *P* < 0.01, and ****P < 0.0001.

To explore the influence of the miR-US25-1 expression on EAhy926 cells, we determined the viability and apoptosis of EAhy926 cells after HCMV infection or miR-US25-1 mimics transfection. The miR-US25-1 mimics significantly promoted the miR-US25-1 level in cells, with a *P* value less than 0.0001 (Figure 2(a)). Firstly, to observe the influence of HCMV infection or miR-US25-1 transfection on the EAhy926 cells viability, the MTT assay was conducted. It was shown in Figure 2(b) that the HCMV infection with a MOI of 1 caused about 30% more cell viability decreasing from 4 to 8 D.P.I. (*P* < 0.05 or *P* < 0.01). And 0.1 MOI infection caused approximately 30% cell viability decreasing at 6 or 8 D.P.I. (*P* < 0.05, either). And the miRNA mimics transfection also significantly reduced the cell viability with a reduction of 20% at 24 H.P.T. or of 25% at 48 H.P.T. (*P* < 0.05, either). To further explore the mechanism of cell viability reduction by miR-US25-1, then, we determined the EAhy926 cell apoptosis after miR-US25-1 mimics transfection. Strikingly, Figures 2(d) and 2(e) demonstrated that there was no significant difference in apoptosis promotion between the miR-US25-1 mimics and miRNA control (*P* > 0.05). And there was also no significant difference in the caspase 3 activity between the miR-US25-1 mimics and miRNA control transfected EAhy926 cells (Figure 2(f)), reconfirming nonpromotion of miR-US25-1 to EAhy926 cell apoptosis.

### 2.3. miR-US25-1 Downregulates the Expression of BRCC 3 by Targeting the 5′ UTR of BRCC 3

**Figure 3:**
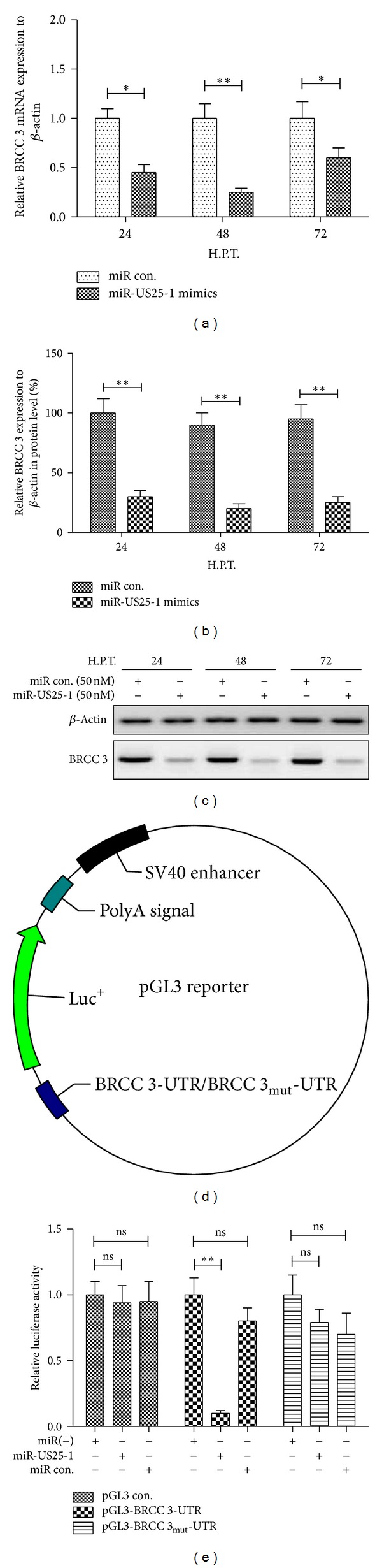
miR-US25-1 downregulated BRCC 3 by targeting the 5′ UTR. (a) BRCC 3 mRNA was reduced in EAhy926 cells after transfection with miR-US25-1 mimics. (b) and (c) Western blot analysis indicated that the expression of BRCC 3 was inhibited in protein level in EAhy926 cells by miR-US25-1 mimics transfection. (d) Schematic diagram of the pGL3-BRCC 3/BRCC 3_mut_ reporter construction. (e) Relative luciferase activity of pGL3-BRCC 3/BRCC 3_mut_ reporter or pGL-con vector in EAhy926 cells transfected with miR-US25-1 mimics. All experiments were performed in triplicate independently. Statistical significance was showed as *P < 0.05 and **P < 0.01, ns: no significance.

Unlike miRNAs encoded by eukaryotic cells, the HCMV-encoded miR-US25-1 was reported to primarily bind sites within the 5′ UTR of targeted genes and mediate the reduction in gene expression in HEK293 cells [45]. And many of the genes targeted by miR-US25-1 are associated with cell cycle control, including cyclin E2 and BRCC3 [45]. To investigate whether there is an association of miR-US25-1-targeted genes with the confirmed viability reduction in EAhy926 cells by miR-US25-1, we determined the expression of BRCC 3, which is one of most regulated molecules by miR-US25-1 and plays a vital role in DNA damage repairing [47] and in mRNA and protein levels in the EAhy926 cells after miR-US25-1 transfection. As shown in Figure 3(a), the miR-US25-1 mimics (50 nM) caused a 50–70% reduction of BRCC 3 mRNA 24–72 h after transfection, compared to the control group (*P* < 0.05 at 24 or 72 h, *P* < 0.01 at 48 h). The western analysis also revealed a significant BRCC 3 reduction (60–80%) in protein level by the miRNA transfection with 50 nM (*P* < 0.05 at 24 and *P* < 0.01 at 48 h or 72 h). To reconfirm the BRCC 3 downregulation by miR-US25-1, we construct a luciferase reporter constructs containing the miR-US25-1 recognizing 5′ UTR sequences of BRCC 3 (Figure 3(d)). As shown in Figure 3(e), 50 nM miR-US25-1 mimics suppressed while the miRNA control did not regulate the activity of pGL3-BRCC 3 reporter in EAhy926 cells (*P* < 0.01); and the miR-US25-1 mimics had no such suppression on the luciferase activity of pGL3-BRCC 3_mut_ or pGL3-con reporter. These results agree with the fact that miR-US25-1 regulates BRCC 3 by targeting the 5′ UTR of it and suppressing its translation.

### 2.4. miR-US25-1 Transfection Deteriorates the ox-LDL-Induced Apoptosis in EAhy926 Cells

**Figure 4:**
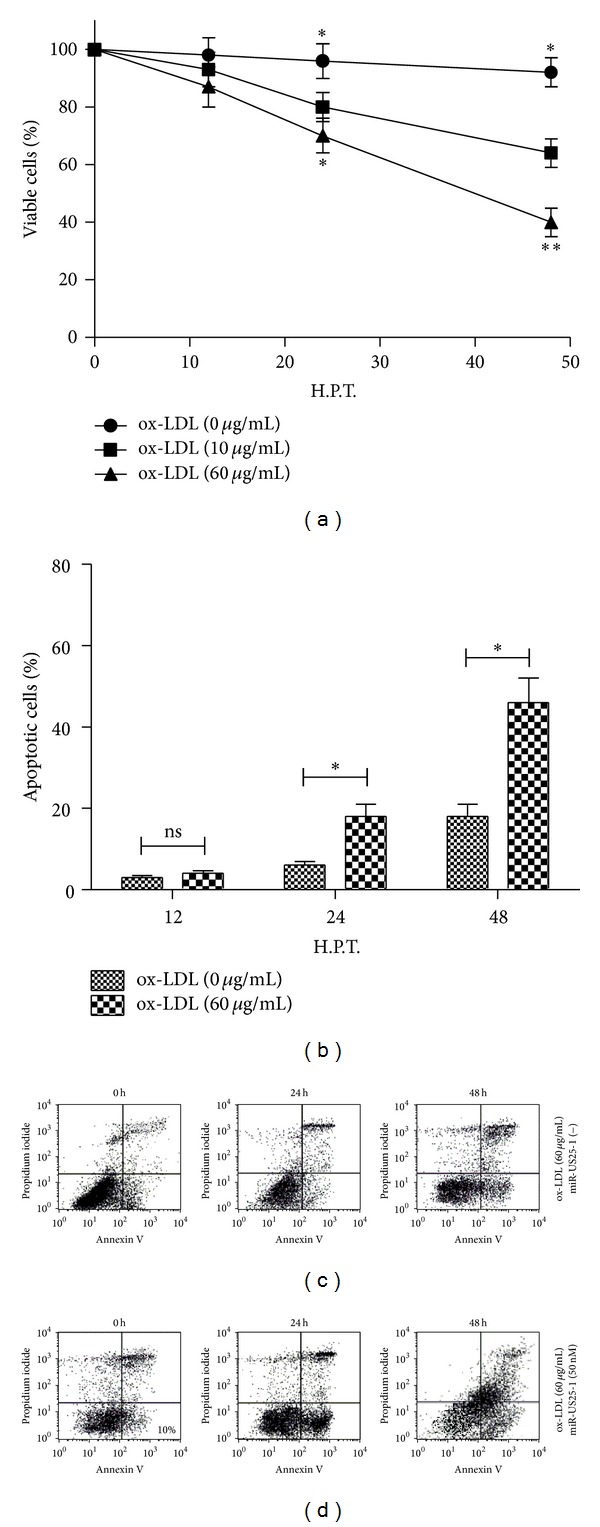
miR-US25-1 aggravates the ox-LDL-induced apoptosis in EAhy926 cells. (a) Viability of EAhy926 cells significantly decreased 24 or 48 h after treatment with 10 or 50 *μ*g/mL ox-LDL (MTT assay). (b) miR-US25-1 mimics aggravated the apoptosis of EAhy926 cells treated with ox-LDL. (c) Apoptosis in EAhy926 cells after 50 *μ*g/mL ox-LDL treatment and 50 nM miRNA control transfection. (d) Apoptosis of EAhy926 cells after treatment of 50 *μ*g/mL ox-LDL and 50 nM miR-US25-1 mimics.

**Figure 5:**
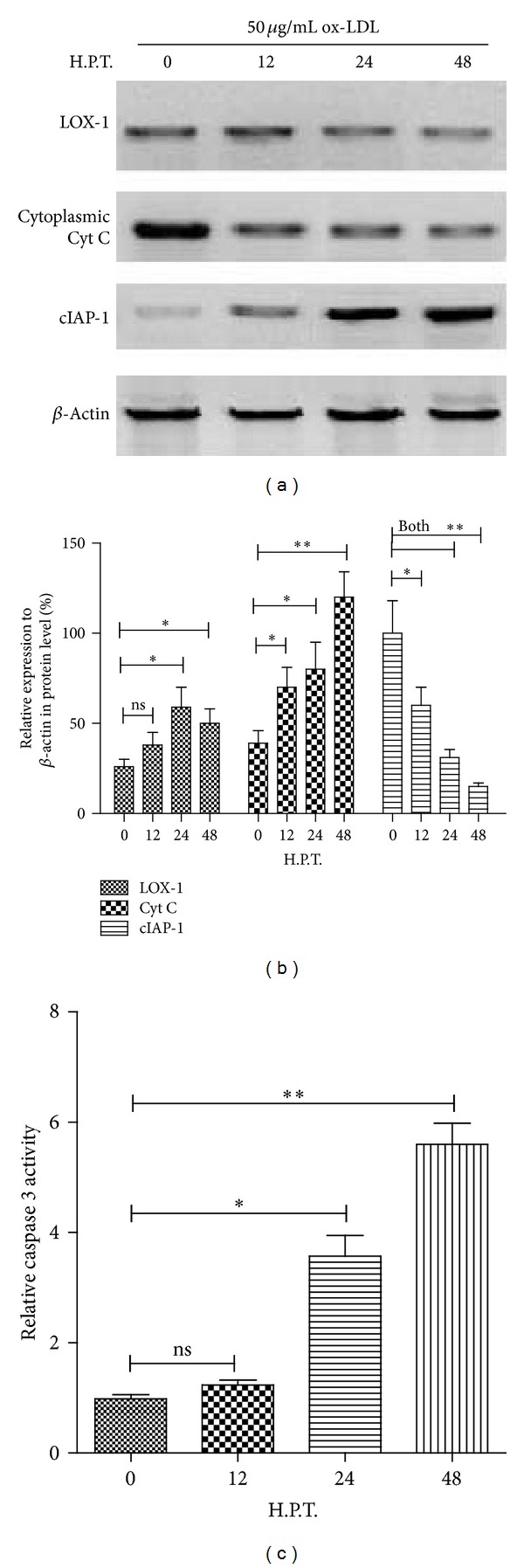
Deregulation of ox-LDL receptor and apoptosis-associated molecules in EAhy926 cells by ox-LDL treatment. (a) and (b) ox-LDL significantly promoted the expression of LOX-1, the ox-LDL receptor on EAhy926 cells, and the release of Cyt c and downregulated cIAP-1 expression, an apoptosis inhibiting protein. Western blot assay was conducted to identify the protein level of the three molecules, compared to *β*-actin. (c) Significant upregulation of caspase 3 activity in EAhy926 cells after treatment of 50 *μ*g/mL ox-LDL. All results were got from triplicate independent experiments. Statistical significance was showed as *P < 0.05 and **P < 0.01, ns: no significance.

HCMV has clearly been shown to be associated with vasculopathy and atherosclerosis [25–28]. And the ox-LDL-induced apoptosis via binding to LOX-1 in vascular endothelial cells [4, 6] has been well determined. To observe the influence of miR-US25-1 on the ox-LDL promoted endothelial cell apoptosis, we treated the EAhy926 cells with ox-LDL and with miR-US25-1 mimics transfection. Firstly, Figure 4(a) showed that the ox-LDL with 10 or 50 *μ*g/mL reduced the EAhy926 cell viability significantly (*P* < 0.05 for 10 *μ*g/mL and for 50 *μ*g/mL at 24 H.P.T.; *P* < 0.01 for 50 *μ*g/mL at 48 h). Then, the cell apoptosis was examined by flow cytometry. It was shown in Figures 4(b)–4(d) that the treatment with ox-LDL induced significantly increased apoptosis (with a percentage of apoptotic cells of 16.32 ± 2.14 at 24 h after treatment and 37.43 ± 5.62 at 48 h after treatment, either *P* < 0.05). And what is more, the miR-US25-1 mimics transfection (50 nM) accelerated the ox-LDL-induced apoptosis (a percentage of 32.67 ± 5.31 versus 16.32 ± 2.14 at 24 H.P.T. and a percentage of 35.58 ± 5.66 versus 56.55 ± 8.19 at 48 H.P.T.; either *P* < 0.05). To further confirm the apoptosis induction by ox-LDL, we quantified by western blot assay the expressions of LOX-1, that is, ox-LDL receptor, cytochrome C (Cyt C) release, and cIAP-1 expression, which were up- or downregulated in the context of ox-LDL-induced apoptosis [6]. It was shown in Figures 5(a) and 5(b) that the ox-LDL induced significantly high level of LOX-1 expression and Cyt C release (*P* < 0.05 for LOX-1 at 24 or 48 h and for Cyt C at 12, 24, or 48 h, resp.). And the caspase 3 activity was also assayed to reconfirm the apoptosis inductivity of ox-LDL.  Figure 5(c) indicated that the caspase 3 activity in EAhy926 cells after ox-LDL treatment was elevated for approximately 4 times at 24 H.P.T. or 6 times at 48 H.P.T., with a significance (*P* < 0.05 or *P* < 0.01). Thus, the apoptosis induction by ox-LDL was confirmed in EAhy926 cells.

**Figure 6:**
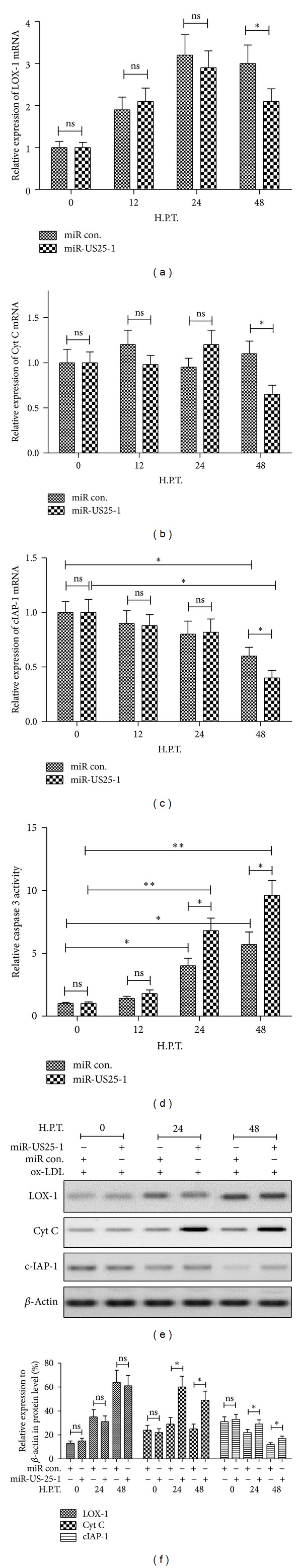
miR-US25-1 aggravated the deregulation of ox-LDL on the expression of LOX and cIAP-1 and the release of Cyt c in EAhy926 cells. (a)–(c) Influence of miR-US25-1 mimics transfection (50 nM) on the expression of LOX, cIAP-1, and Cyt c in mRNA levels in EAhy926 cells after 50 *μ*g/mL ox-LDL treatment. (d) miR-US25-1 mimics transfection (50 nM) deteriorated the upregulation of the caspase 3 activity in EAhy926 cells by treatment of 50 *μ*g/mL ox-LDL. (e) and (f) Western blot assay of LOX expression, cIAP-1 expression, and Cyt c release in EAhy926 cells which were treated with 50 *μ*g/mL ox-LDL and transfected with 50 nM miR-US25-1 mimics or 50 nM miRNA control (*β*-actin as control). All results were got from triplicate independent experiments. Statistical significance was showed as *P < 0.05 and **P < 0.01, ns: no significance.

An aggravation to the ox-LDL-induced apoptosis of miR-US25-1 has been confirmed by the flow cytometry for apoptotic cells. To further confirm the aggravation by miR-US25-1 in the molecular level, the regulation of miR-US25-1 on LOX-1, Cyt C, and cIAP-1 was assayed in both mRNA and protein levels. It was shown that the miR-US25-1 did not downregulate the expression of LOX-1, Cyt C, and cIAP-1 in mRNA level (Figures 6(a)–6(c)) before 48 hours after transfection. However, at 48 H.P.T., mRNAs of the three molecules were expressed in a significantly low level in the miR-US25-1-transfected cells than in the control miRNA-transfected cells; particularly, the cIAP-1 mRNA even reduced in cells transfected control microRNA (Figures 6(a)–6(c)). Then, the caspase 3 activity was also assayed to identify the aggravation by miR-US25-1 on the apoptosis inductivity by ox-LDL.  Figure 6(d) indicated that the caspase 3 activity in EAhy926 cells after miR-US25-1 transfection was elevated significantly higher than in the cells after control miRNA transfection, either at 24 or 48 H.P.T. (*P* < 0.05 either). Western blot analysis was also conducted to analyze the influence of miR-US25-1 on apoptosis-associated molecules. It was shown that the upregulation by ox-LDL in LOX-1 expression was not significantly influenced by the miR-US25-1, compared to the control microRNA group (*P* > 0.05 for 24 or 48 H.P.T.). However, the miR-US25-1 aggravated the promotion by ox-LDL on the Cyt C release (a percentage of 61.50 ± 7.85 versus 29.52 ± 4.26 at 24 H.P.T. and a percentage of 52.25 ± 7.33 versus 24.32 ± 4.50 at 48 H.P.T.; either *P* < 0.05) and c-IAP-1 expression (a percentage of 29.80 ± 4.23 versus 19.32 ± 2.98 at 24 H.P.T. and a percentage of 44.47 ± 5.75 versus 32.85 ± 4.25 at 48 H.P.T.; either *P* < 0.05).

## 3. Discussion

Studies have demonstrated that the HCMV genomes, as other members of its Herpesviridae family, encode miRNAs [42, 43, 48], which were shown to participate in the complex regulation of host cell metabolism and immune evasion [39, 44, 49]. HCMV miRNAs have been demonstrated to be expressed during acute lytic infection [40–43]. miR-US25-1 is one of HCMV-encoded miRNAs and targets cellular genes that are essential for virus growth to control the life cycle of the virus [44]. More recently, it is shown that the viral miR-US25-1 downregulates multiple cell cycle genes through mRNA 5′ UTRs [45]. The prominent regulation of cell cycle genes of the miR-US25-1 attracts us to explore its role in the atherosclerosis promotion.

In present study, we confirmed the upregulation of miR-US25-1 level in normal subjects or atherosclerosis patients and in endothelial cells with HCMV infection; there was a significant association of miR-US25-1 level with the active HCMV infection, and the miR-US25-1 expression correlated with the mRNA level of pp65, one of HCMV tegument proteins in the atherosclerosis patients. The* in vitro* experiment also revealed that HCMV infection promoted miR-US25-1 expression in EAhy926 endothelial cells. Then the regulatory role of miR-US25-1 in EAhy926 cells was determined. It was shown that the miR-US25-1 mimics reduced cell viability but did not induce apoptosis in EAhy926 cells.

It has been reported that the HCMV-encoded miR-US25-1 primarily binds sites within the 5′ UTR of targeted genes and mediates the reduction in gene expression [45]. And many of the targeted genes are associated with cell cycle control, including cyclin E2 and BRCC3 [45]. To explore the mechanism into the confirmed viability reduction by miR-US25-1, we investigated the association of miR-US25-1 with its possible targeted genes, involving cyclin E2, BRCC3, and EID1, and found a significant reduction of BRCC 3 in EAhy926 cells after miR-US25-1 mimics transfection, while no significant results got in cyclin E2 and EID1 mRNA expression (data not shown). Since BRCC 3 is one of most regulated molecules by miR-US25-1, we speculated that the cell viability reduction might be caused by the BRCC 3 blockage. And to further investigate the role of miR-US25-1 in the HCMV-associated atherosclerosis, we then determined the synergy role of miR-US25-1 in the ox-LDL-induced apoptosis in EAhy926 cells, given the vital role of BRCC 3 in DNA damage repairing [47].

Firstly, we confirmed that the ox-LDL reduced the viability of EAhy926 cells significantly via inducing cell apoptosis (flow cytometry with annexin V/PI double staining). The western blot results reconfirmed the on-LDL-promoted apoptosis, revealing by the upregulation of LOX-1 expression and cytochrome C (Cyt C) release the downregulation of cIAP-1 expression in the context of ox-LDL-induced apoptosis [6]. And the caspase 3 activity was also significantly promoted by ox-LDL. Thus, the apoptosis induction by ox-LDL was confirmed in EAhy926 cells. And what is more, the miR-US25-1 mimics transfection accelerated the ox-LDL-induced apoptosis by both flow cytometry assay and western blot analysis of apoptosis-associated molecules.

Life-long persistent HCMV infection is linked to the development and severity of the cardiovascular disease atherosclerosis [24], with a serological confirmation [27, 28]. And* in vivo* studies have revealed that HCMV infection of the vessel wall affects various cells including monocytes/macrophages, smooth muscle cells (SMCs), and endothelial cells (ECs) leading to the dysfunction of ECs and the activation of proinflammatory signaling [30], which promotes enhanced proliferation, migration of monocytes and SMCs into the intima of the vascular wall, and expansion of atherosclerotic lesion [50, 51]. An enhanced expression of endothelial adhesion molecules has also been promoted by HCMV infection [30], as a consequence of which infected endothelium recruits naive monocytes in the infected sites. Endothelial damage by HCMV infection may also promote thrombin generation linking inflammation and coagulation [52]. However, there is little direct evidence of the association of HCMV with the atherosclerosis. Present study indicated that the HCMV-encoded microRNA and miR-US25-1 aggravated the ox-LDL-induced apoptosis of endothelial cells via targeting and downregulating BRCC 3; the synergy role of it in ox-LDL-induced apoptosis might contribute to the association of HCMV with atherosclerosis.

miR-US25-1 was reported to primarily bind sites within the 5′ UTR of targeted genes and mediate the reduction in gene expression [45]. And many of the genes targeted by miR-US25-1 are associated with cell cycle control, including cyclin E2 and BRCC3 [45]. In the present study, we have also confirmed the deregulation of BRCC 3 expression by miR-US25-1 and targeting the 5′ UTR of BRCC 3. Given the vital role of BRCC 3 in DNA damage repairing [47], we speculated that the targeting inhibition of BRCC 3 by miR-US25-1 may contribute to the cell viability reduction and the aggravation to ox-LDL-promoted apoptosis of endothelial EAhy926 cells.

## 4. Material and Methods

### 4.1. Subjects

A group of 28 subjects with HCMV infection and 10 subjects without HCMV infection were recruited in this study from the outpatients of the Second Xiangya Hospital of Central South University. All atherosclerosis patients with (32 cases) or without (15 cases) HCMV infection were selected from the outpatients or inpatients registered in Cardiovascular Medicine Department, the Second Xiangya Hospital of Central South University. Informed consent was obtained from all subjects, and the study was approved by the Ethics Committee of Central South University.

### 4.2. Cell Cultures, Virus, and Reagents

The human embryo fibroblast cells (HEF cells) were utilized for HCMV isolation and growth determination, and the human umbilical vein cell line, EAhy926 cells were utilized for HCMV growth curve assay. HEF cells and EAhy926 cells were provided by the Central Laboratory, Central South University, and cultured in DMEM or RPMI 1640 medium (Invitrogen, Carlsbad, CA, USA) supplemented with 10% FBS (Gibco, Rockville, MD, USA) at 37°C under 5% CO_2_. The DMEM or RPMI 1640 medium supplemented with 2% FBS was used for the HEF cells or EAhy926 cells maintaining postgrowing to about 90% confluence.

HCMV strain, AD169, was used to determine the virus growth curve and miR-US25-1 promotion with 0.1 or 1 MOI of AD169. ox-LDL (Cat.# BT-910) was purchased from Biomedical Technologies Inc. (Stoughton, MA, USA) and resolved in the RPMI 1640 medium supplemented with 2% FBS. ox-LDL with a concentration of 50 *μ*g/mL was used for EAhy926 cell treatment. The miR-US25-1 mimics (GenePharma, Shanghai, China) were utilized to manipulate the miR-US25-1 level in EAhy926 cells. 50 nM miRNA mimics or miRNA control (scramble-control miRNA) (GenePharma, Shanghai, China) was transfected with Lipofectamine 2000. To identify the influence of miR-US25-1 on the cell viability or apoptosis, the EAhy926 cells after miR-US25-1 mimics or miRNA control transfection for various hours or days was maintained, with or without ox-LDL treatment.

### 4.3. RNA Isolation, Reverse Transcription, and qPCR

Total cellular RNA was purified with PureLink RNA Mini Kit (Invitrogen, Carlsbad, CA, USA), and miRNAs were isolated using mirVana miRNA Isolation Kit (Ambion, Austin, TX, USA) according to the kit manuals. The quantitative reverse transcription-PCR (RT-PCR) was performed with Takara One Step RT-PCT kit (Takara, Dalian, China). For quantitative analysis of mRNA expression of pp65 of HCMV, LOX-1, Cyt c, and c-IAP-1, the mRNA samples were amplified using primer/probe sets specific for the genes of interest on a Lightcycler 480 II (Roche, Mannheim, Germany). Relative quantification was determined using the ΔΔCt method using *β*-actin as reference gene [53]. The primers used were available upon request.

### 4.4. Plaque Forming Assay and Growth Curve

Cell-free HCMV virus stocks (AD169) were serially 10-fold diluted in DMEM medium with 2% FBS and inoculated HEF monolayer cells for 2 hours; then the cell layers were washed with warm PBS twice and overlaid with 1% methylcellulose. After incubation for 7 to 10 days, the cultures were fixed and plaques were stained with 0.5% crystal violet. PFU per milliliter counted. HEF or EAhy926 cells were cultured in growth plate with 12 holes to 90% confluence and were inoculated with 0.1 MOI AD169 virus for 2 hours. Cells were replaced with new medium and incubated for various time, after three-time washing with PBS. Then the cell supernatant was collected and titered by plaque forming assay.

### 4.5. Cell Viability and Apoptosis Assays

Cell viability was evaluated by methyl thiazolyl tetrazolium assay (MTT assay) (Invitrogen, Carlsbad, CA, USA). EAhy926 cells after (both) ox-LDL treatment or (and) miR-US25-1 transfection were incubated with 50 *μ*L MTT solution at 37°C for another 2 h and were dissolved by 150 *μ*L DMSO completely at room temperature. The absorbance was then measured at 570 nm using a spectrophotometer (Bio-Rad, Hercules, CA, USA). Cell apoptosis was determined by an annexin V-FITC apoptosis detection kit (Sigma-Aldrich, St. Louis, MO, USA). Briefly, 1–5 × 10^5^ cells after ox-LDL treatment or (and) miR-US25-1 transfection were collected and resuspended in 0.5 mL of binding buffer and incubated with annexin V-FITC and propidium iodide for 10 min in the dark at room temperature and then assayed with an FACScan flow cytometer (Bio-Rad, Hercules, CA, USA) equipped with a FITC signal detector FL1 (excitation 488 nm, green) and a phycoerythrin emission signal detector FL3 (excitation 585 nm, red). Results were calculated using the CellQuest Pro software (BD Biosciences) and expressed as the percentage of apoptotic (including early and late apoptotic cells) to total cells. Caspase 3 activity was determined using the Enzchek Caspase-3 Assay Kit #2 (Invitrogen). Cell extracts were incubated with the caspase substrate Z-DEVD-R110 (25 mM, final concentration) for 3.5 h in the dark, at room temperature. The appearance of fluorescent rhodamine 110 (R-110) upon enzymatic cleavage of the nonfluorescent substrate Z-DEVD-R110 was subsequently assayed using a microplate reader (Ex: 485 nm; Em: 520 nm). Negative controls (samples without the Z-DEVD-R110 substrate) and substrate-only controls (mixture of activity buffer and Z-DEVD-R110 substrate to determine the background fluorescence of the substrate) were also carried out. The results were expressed as a relative value to the caspase 3 activity of pretreated cells.

### 4.6. Immunoblotting

Cells (approximately 10^5^) were lysed with the Cytoplasmic, Mitochondria, Membrane, and Nuclear Protein Extraction Kit (ZmTech Scientific Inc., San Jose, CA, USA) and supplemented with Complete Mini Protease Inhibitor Cocktail (Roche, Basel, Switzerland) according to manuals. After protein concentration determination using Bradford Reagent (Bio-Rad, Hercules, CA, USA), the protein samples were separated by a 12% gradient SDS-PAGE gel, transferred to PVDF membrane, and blocked in 5% skimmed milk. The following primary antibodies were utilized: BRCC 3 rabbit polyclonal antibody, 1 : 300 (Santa Cruz Biotech), LOX-1 rabbit polyclonal antibody, 1 : 300 (Sino Biological, Beijing, China), Cyt c rabbit polyclonal antibody, 1 : 500 (Sigma-Aldrich, St. Louis, MO, USA), c-IAP-1 rabbit polyclonal, 1 : 500 (Santa Cruz Biotechnology, Santa Cruz, CA, USA), or *β*-actin rabbit polyclonal, 1 : 1000 (Sigma-Aldrich, St. Louis, MO, USA). Goat anti-rabbit IgG conjugated to horseradish peroxidase (Pierce, Rockford, IL, USA) and ECL detection systems (Super Signal West Femto; Pierce, Rockford, IL, USA) were used for detection.

### 4.7. Luciferase Activity Assay

Reporter plasmid with BRCC 3 or BRCC 3 mut 5′ UTR (pGL3) was constructed by our lab; the pGL3 with the 5′ UTR of BRCC 3 was inserted with the miR-US25-1 targeting sequence in BRCC 3 (UGGCGGU) with three repeated copies, at the multiple cloning sites before luciferase coding sequence. While the inserted sequence in the pGL3 with the mutated 5′ UTR of BRCC 3 was mutated to cGaataac 1 × 10^5^, EAhy926 cells were transfected with 50 nM of miRNA control or miR-US25-1 mimics and 0.5 *μ*g reporter plasmid with Lipofectamine 2000. 6 h after transfection, cells were inoculated with RPMI 1640 containing 10% FBS for another 24 h and harvested for analysis. Luciferase activity was determined with a Promega Luciferase Assay System (Madison, WI, USA).
